# Genetic variation and cognitive dysfunction in opioid‐treated patients with cancer

**DOI:** 10.1002/brb3.471

**Published:** 2016-05-03

**Authors:** Geana Paula Kurita, Ola Ekholm, Stein Kaasa, Pål Klepstad, Frank Skorpen, Per Sjøgren

**Affiliations:** ^1^ Multidisciplinary Pain CentreDepartment of NeuroanaesthesiologyRigshospitalet Copenhagen University HospitalCopenhagenDenmark; ^2^ Department of OncologyRigshospitalet Copenhagen University HospitalCopenhagenDenmark; ^3^ National Institute of Public HealthUniversity of Southern DenmarkCopenhagenDenmark; ^4^ Department of OncologyOslo University Hospital/University of OsloNorway; ^5^ European Palliative Care Research CentreFaculty of Medicine Norwegian University of Science and TechnologyTrondheimNorway; ^6^ Department of Intensive Care MedicineSt Olavs HospitalTrondheim University HospitalTrondheimNorway; ^7^ Department of Circulation and Medical ImagingNorwegian University of Science and TechnologyNorway; ^8^ Department of Laboratory MedicineChildren's and Women's HealthNorwegian University of Science and TechnologyTrondheimNorway; ^9^ Section of Palliative MedicineDepartment of OncologyRigshospitalet Copenhagen University HospitalCopenhagenDenmark; ^10^ Department of Clinical MedicineFaculty of Health and Medical SciencesUniversity of CopenhagenCopenhagenDenmark

**Keywords:** Cognition, genes, neoplasms, opioids, polymorphism, single nucleotide

## Abstract

**Background and purpose:**

The effects of single‐nucleotide polymorphisms (SNPs) on the cognitive function of opioid‐treated patients with cancer until now have not been explored, but they could potentially be related to poor functioning. This study aimed at identifying associations between SNPs of candidate genes, high opioid dose, and cognitive dysfunction.

**Methods:**

Cross‐sectional multicenter study (European Pharmacogenetic Opioid Study, 2005–2008); 1586 patients; 113 SNPs from 41 genes. Inclusion criteria: cancer, age ≥18 year, opioid treatment, and available genetic data. Cognitive assessment by Mini‐Mental State Examination (MMSE). Analyses: SNPs were rejected if violation of Hardy–Weinberg equilibrium (*P* < 0.0005), or minor allele frequency <5%; patients were randomly divided into discovery sample (2/3 for screening) and validation sample (1/3 for confirmatory test); false discovery rate of 10% for determining associations (Benjamini–Hochberg method). Co‐dominant, dominant, and recessive models were analyzed by Kruskal–Wallis and Mann–Whitney tests.

**Results:**

In the co‐dominant model significant associations (*P* < 0.05) between MMSE scores and SNPs in the *HTR3E*,*TACR1*, and *IL6* were observed in the discovery sample, but the replication in the validation sample did not confirm it. Associations between MMSE scores among patients receiving ≥400 mg morphine equivalent dose/day and SNPs in *TNFRSF1B*,*TLR5*,*HTR2A*, and *ADRA2A* were observed, but they could not be confirmed in the validation sample. After correction for multiple testing, no SNPs were significant in the discovery sample. Dominant and recessive models also did not confirm significant associations.

**Conclusions:**

The findings did not support influence of those SNPs analyzed to explain cognitive dysfunction in opioid‐treated patients with cancer.

## Introduction

Patients with advanced cancer develop very frequently a wide range of symptoms, including cognitive dysfunction, which interfere with their daily life, health status, prognosis, compliance to treatment, social interactions, and quality of life. Causes for development of cognitive alterations are multiple and may be attributed to the cancer disease itself, comorbidities, and treatments including opioid therapy (Levine et al. 1978; Massie et al. 1983; Sjøgren 1997; Bruera et al. 1992; Baumgartner 2004; Kurita and Pimenta 2008). Some causes may be reversible or manageable; however, the knowledge and scientific exploration regarding this issue in patients with cancer is in its infancy.

Opioid treatment to manage cancer pain is the cornerstone in clinical practice and these drugs are highly recommended by WHO (1996) for this purpose. However, opioids have several adverse effects on the central nervous system and many of these effects are still unclear. Opioids can interfere with acquirement, processing, storage, and retrieval of information (Lawlor 2002). In addition to altering cognitive processes associated with memory, they can alter psychomotor function, mood, concentration, and other mental capabilities (Kurita et al. 2009).

In the past, questions related to opioid effects on cognition in patients with cancer did not represent a major point of concern. In palliative care, a possible reason for this was due to fast disease progression and short life expectancy. However, recently, an increased attention regarding cognitive functioning in palliative care as well as during the entire cancer trajectory has been noticed, although neuropsychological assessment of patients with cancer is a relatively new research area still based on rather limited scientific evidence. Thus, identification of mental alterations, specially mild and subtle alterations, are still frequently ignored and left undisclosed and treated (Inouye et al. 2001; Pisani et al. 2003).

We have formerly undertaken two studies in a multinational sample of opioid‐treated patients with cancer, in which the cognitive effects of a wide range of variables were investigated (Kurita et al. 2011, 2015). They demonstrated that nearly 1/3 of opioid‐treated patients with cancer presented possible or definite cognitive dysfunction and several factors, including opioid dose, were associated with the dysfunction (Kurita et al. 2011, 2015). Based on these series of studies, we considered that genetic factors could also be involved in the cognitive performance of opioid‐treated patients with cancer and decided to proceed with analyzing potential candidate genes in the sample investigated in the previously mentioned studies.

Literature on associations between cognitive dysfunction and genetic variation in opioid‐treated patients with cancer is practically nonexistent. In addition, knowledge on genetic influence on some specific cognitive disorders seems to be sparse (Flint 1999, 2001). Therefore, this study aimed at analyzing associations between single‐nucleotide polymorphisms (SNPs) of candidate genes and cognitive functioning in opioid‐treated patients with cancer. Moreover, keeping in mind that high opioid doses have previously been associated with cognitive dysfunction (Kurita et al. 2011, 2015), associations between SNPs in patients treated with high opioid doses and cognitive functioning were also investigated.

## Methods

### Design and sample

The sample analyzed in this study is derived from the European Pharmacogenetic Opioid Study (EPOS), which is a cross‐sectional and multicenter investigation conducted in 11 countries during 2005–2008 (Klepstad et al. 2011). The original sample was composed by 2294 patients with cancer pain who were ≥18 year of age, had regular opioid treatment for at least 3 days for moderate or severe pain and able to speak the language used at the study center. In this study, we selected those with available genetic data and cognitive assessment by Mini‐Mental State Examination (MMSE).

Research protocol was approved by local ethics committees (Regional Medical Research Ethics Committee, Central Norway Health Authority, Protocol reference number: 119‐03, approved 27.09.03) and conducted in accordance with ethical standards of the Declaration of Helsinki. Written informed consent was obtained from all patients prior to their inclusion in the study.

### Genotyping procedures

Blood samples were collected from the patients, handled, and stored in each center according to the study protocol, before shipment to the Department of Laboratory Medicine, Children's and Women's Health, Faculty of Medicine, Norwegian University of Science and Technology, where the genotyping analyses took place. DNA was extracted from EDTA‐blood using the Gentra Puregene blood kit (Qiagen Science, Germantown, MD). Genotyping was performed by the SNPlex Genotyping System according to the supplier's dry DNA protocol (Applied Biosystems, Foster City, CA). Capillary electrophoresis was carried out on an ABI 3730 48‐capillary DNA analyzer (Applied Biosystems). SNPlex signals were analyzed using the Gene Mapper v.4.0 software (Applied Biosystems) followed by manual reading. Samples with signals that could not be discriminated from those of negative controls were excluded and treated as missing data. Two SNPs, rs4680 and rs1045642, were genotyped by TaqMan SNP allelic discrimination analysis, using an ABI 7900HT analyzer (Applied Biosystems).

In this study, selection of candidate genes and SNPs was restricted to a previous pool of genes analyzed regarding genetic variations and morphine efficacy (Klepstad et al. 2011). Those genes in the pool that according to the literature had any relation to cognitive function were selected for the present analyses.

### Cognitive function assessment

Mini‐Mental State Examination is an observer‐rated brief battery of simple cognitive tests, which measure orientation to time and place, registration of words, attention, calculation, word recall, language, and visual construction. Scores range from 0 to 30. The cutoff between scores 26 and 24 means possible cognitive dysfunction and below 24 definite cognitive dysfunction (Folstein et al. 1984; Crum et al. 1993; Kurita et al. 2011).

### Analyses

Statistical analyses were performed based on four steps:

1. The candidate SNPs were rejected if there was evidence of violation of Hardy–Weinberg equilibrium, which in the present data set was calculated as the difference between the observed and expected frequencies being *P* < 0.0005. They were also rejected if the minor allele frequency was <5%.
2. Patients were randomly divided into discovery sample for initial SNPs screening (discovery phase: 2/3 patients) and the validation sample for confirmatory test (replication phase: 1/3 patients). In order to confirm that SNPs is associated with cognitive function, the significant results found in the discovery sample should be replicated in the validation sample.
3. A false discovery rate of 10% was used for determining associations (Benjamini–Hochberg method), in which 10% of the positive results were expected to be false positives (Benjamini and Hochberg 1995).
4. The model chosen for the primary genetic analysis was the co‐dominant model and associations were analyzed considering MMSE scores as a continuous variable and applying Kruskal–Wallis test. Secondary analyses were performed using dominant and recessive models, in which Mann–Whitney test was used. In addition, opioid daily doses were converted to equipotent mg of oral morphine as described in a previous study (Kurita et al. 2011) and further analyses were performed considering only patients receiving ≥400 mg morphine equivalent dose/day due to the fact that association between cognitive dysfunction and opioid dose at this level was observed (Kurita et al. 2011). *P*‐values below 0.05 were considered significant.

## Results

### Sample characteristics

A total of 1586 patients were analyzed. However, patients with missing MMSE scores were excluded (*n* = 217). Most of them were patients from Norway (24.0%), Italy (19.9%), Germany (17.4%), and United Kingdom (17.2%). There were equal proportions of men (50.1%) and women (49.9%) and the majority were between 50 and 79 years old (76.5%). Approximately 80% of the sample was composed of inpatients, 23.8% were being treated with ≥400 mg morphine equivalent dose/day and 27.6% had possible or definite cognitive dysfunction (Table 1).

**Table 1:** Patient's characteristics (*n* = 1586)

Characteristics	*n*	%
Country of residence		
Denmark	19	1.2
Finland	22	1.4
Germany	276	17.4
Greece	3	0.2
Iceland	108	6.8
Italy	316	19.9
Lithuania	35	2.2
Norway	380	24.0
Sweden	91	5.7
Switzerland	64	4.0
United Kingdom	272	17.2
Gender		
Men	795	50.1
Women	791	49.9
Age		
18–39 year	76	4.8
40–49 year	185	11.7
50–59 year	352	22.2
60–69 year	491	31.0
70–79 year	371	23.4
≥80 year	110	6.9
No information	1	0.1
Settings		
Palliative care unit /Hospice	535	33.7
General oncology ward	645	40.7
Surgical ward	59	3.7
Outpatient clinic	347	21.9
Cancer type		
GI	300	18.9
Lung	233	14.7
Breast	214	13.5
Prostate	172	10.8
Female reproductive organs	113	7.1
Urologic	103	6.5
Hematologic	94	5.9
Head and neck	62	3.9
Sarcoma	41	2.6
Pancreatic	32	2.0
Skin	25	1.6
Other or more than one type	197	12.4
Metastasis CNS		
Yes	97	6.1
No	1489	93.9
Karnofsky performance		
Able to carry on normal activity/work	343	21.6
Unable to work	932	58.8
Unable to care for self	308	19.4
No information	3	0.2
Type of opioid		
Morphine only	610	38.5
Fentanyl only	405	25.5
Oxycodone only	272	17.2
Hydromorphone only	54	3.4
Buprenorphine	36	2.3
Methadone	30	1.9
Other or combination of opioids	178	11.2
No information	1	0.1
Opioid mg/day (morphine eq.)		
<400	1209	76.2
≥400	377	23.8
Mini Mental State Examination score		
≤26	437	27.6
>26	932	58.8
No information	217	13.6

### Candidate genes

Forty‐one candidate genes and 113 SNPs were analyzed. Out of them, six genes were excluded because they violated Hardy–Weinberg equilibrium or the minor allele presented a very low frequency. In addition, SNPs with more than 25% missing values were excluded from all analyses. Finally, 83 SNPs in 35 genes were analyzed in 1369 patients (Table 2).

**Table 2:** Candidate genes (*n* = 1586 patients)

Gene (gene product)	Link	Polymorphism	Alleles	Genotypes; *n* (%)		
*COMT* (catechol‐*O*‐methyl transferase)	Cognitive decline in late life (Fiocco et al. 2010)	rs5993882	T>G	772 (58.8)	469 (35.7)	71 (5.4)
		rs4646312	T>C	467 (35.7)	630 (48.2)	210 (16.1)
		rs4680	A>G	360 (26.8)	683 (50.9)	299 (22.3)
		rs2020917^a^				
*ADRA2A* (adrenoceptor alpha 2A)	Attention deficit in children (Gizer et al. 2009)	rs11195419	C>A	963 (77.9)	253 (20.5)	20 (1.6)
		rs553668^a^				
*TACR1* (tachykinin receptor 1)	Inattentiveness in mice (Yan et al. 2011)	rs881	G>C	911 (68.8)	366 (27.6)	47 (3.5)
		rs4439987	A>G	365 (28.7)	638 (50.1)	270 (21.2)
		rs2160652	G>T	609 (46.2)	541 (41.0)	168 (12.7)
		rs12475818	G>T	345 (27.3)	591 (46.8)	327 (25.9)
		rs3771836	G>T	354 (27.8)	614 (48.3)	304 (23.9)
		rs10191107	A>G	413 (32.2)	603 (47.0)	268 (20.9)
		rs12713837	G>C	478 (37.3)	607 (47.4)	196 (15.3)
		rs6725334	A>G	313 (25.8)	611 (50.4)	289 (23.8)
*DRD2* (dopamine receptor D2)	Attention deficit in children (Gizer et al. 2009)	rs1554929	A>G	377 (30.3)	605 (48.6)	262 (21.1)
		rs6279	G>C	598 (45.8)	563 (43.1)	144 (11.0)
		rs1125394	A>G	979 (74.8)	308 (23.5)	21 (1.6)
		rs17601612	G>C	462 (36.1)	613 (48.1)	200 (15.7)
		rs4274224	A>G	323 (25.5)	655 (51.6)	291 (22.9)
		rs7131056	C>A	430 (34.0)	623 (49.3)	211 (16.7)
		rs4648317	C>T	917 (72.5)	318 (25.1)	30 (2.4)
		rs1800496^b^, rs7131440^a^				
*DRD3* (dopamine receptor D3)	Attention deficit in children (Gizer et al. 2009)	rs9817063	T>C	395 (29.6)	666 (49.9)	275 (20.6)
		rs963468	G>A	525 (39.8)	604 (45.8)	190 (14.4)
		rs167771	A>G	878 (66.2)	397 (29.9)	51 (3.8)
		rs324026	T>C	545 (44.1)	536 (43.4)	154 (12.5)
*HTR1A* (5‐hydroxytryptamine (serotonin) receptor 1A, G protein‐coupled)	Learning and memory (Meneses 1999)	rs878567^a^				
*HTR2A* (5‐hydroxytryptamine (serotonin) receptor 2A, G protein‐coupled)	Learning and memory (Meneses 1999)	rs6311	C>T	424 (32.2)	635 (48.3)	257 (19.5)
		rs6312	A>G	1138 (87.4)	157 (12.1)	7 (0.5)
*HTR3A* (5‐hydroxytryptamine (serotonin) receptor 3A, ionotropic	Learning and memory (Meneses 1999)	rs1062613^a^, rs2276302^a^, rs1176719^a^, rs1176713^a^				
*HTR3B* (5‐hydroxytryptamine (serotonin) receptor 3B, ionotropic)	Learning and memory (Meneses 1999)	rs11214763	G>A	877 (70.6)	331 (26.7)	34 (2.7)
		rs1672717	T>C	461 (35.9)	628 (48.9)	196 (15.3)
		rs7943062	G>A	897 (70.2)	348 (27.2)	33 (2.6)
		rs1176744	T>G	549 (44.2)	579 (46.6)	114 (9.2)
		rs2276307^a^, rs3782025^a^				
*HTR3C* (5‐hydroxytryptamine (serotonin) receptor 3C, ionotropic)	Learning and memory (Meneses 1999)	rs6766410^a^, rs6807362^a^, rs6807670^a^, rs6808122^a^				
*HTR3D* (5‐hydroxytryptamine (serotonin) receptor 3D, ionotropic)	Learning and memory (Meneses 1999)	rs6792482, rs939334^a^, rs7621975^a^	T>C	386 (31.1)	628 (50.6)	227 (18.3)
*HTR3E* (5‐hydroxytryptamine (serotonin) receptor 3E, ionotropic)	Learning and memory (Meneses 1999)	rs6443950	T>A	528 (40.1)	629 (47.8)	160 (12.1)
		rs7627615^a^, rs4912522^a^				
*HTR4* (5‐hydroxytryptamine (serotonin) receptor 4, G protein‐coupled)	Learning and memory (Meneses 1999)	rs4264931	G>A	428 (32.5)	656 (49.8)	233 (17.7)
		rs1971431^a^, rs2068190^a^, rs1862342^a^				
*HRH1* (histamine receptor H1)	Learning and memory in mice (Dai et al. 2007)	rs2606731^a^, rs346070^a^				
*ADRB1* (adrenoceptor beta 1)	Alzheimer disease (Bullido et al. 2004)	rs1801253^a^				
*ADRB2* (adrenoceptor beta 2)	IQ, memory and learning in young and elderly (Bochdanovits et al. 2009)	rs1042713	G>A	493 (39.4)	578 (46.2)	179 (14.3)
		rs1042714	C>G	428 (34.0)	600 (47.7)	231 (18.3)
		rs1042717	G>A	788 (64.5)	374 (30.6)	59 (4.8)
		rs1800888^b^, rs1042719^a^				
*GABBR2* (gamma‐aminobutyric acid (GABA) B receptor, 2)	Epilepsy (Wang et al. 2008)	rs10818743	T>G	858 (67.8)	378 (29.9)	29 (2.3)
		rs2304389	G>A	918 (71.4)	327 (25.4)	41 (3.2)
		rs1435252	C>T	625 (49.2)	534 (42.0)	111 (8.7)
		rs2779562	T>C	321 (25.0)	646 (50.4)	315 (24.6)
		rs2808536	C>A	591 (49.3)	516 (43.1)	91 (7.6)
		rs570138	C>T	787 (61.6)	424 (33.2)	67 (5.2)
		rs3750344	A>G	848 (67.4)	248 (19.7)	162 (12.9)
*IL1R1* (interleukin 1 receptor, type I)	General cognitive performance (Benke et al. 2011)	rs2228139	C>G	1110 (88.3)	141 (11.2)	6 (0.5)
*IL1A* (interleukin 1 alpha)	General cognitive performance (Benke et al. 2011)	rs17561	G>T	642 (50.5)	515 (40.5)	114 (9.0)
*IL1B* (interleukin 1 beta)	General cognitive performance (Benke et al. 2011; Sasayama et al. 2011)	rs1143634	C>T	711 (56.6)	464 (36.9)	82 (6.5)
		rs1143627	T>C	551 (43.3)	577 (45.3)	145 (11.4)
*IL4* (interleukin 4)	Inflammation impact on cognitive function (Gorelick 2010; Simen et al. 2011; Goldstein et al. 2014)	rs2243248	T>G	1109 (86.9)	156 (12.2)	11 (0.9)
		rs2070874	C>T	877 (70.0)	344 (27.5)	32 (2.6)
*IL6* (interleukin 6)	Delirium (van Munster et al. 2011)	rs2069835	T>C	1062 (86.6)	155 (12.6)	9 (0.7)
		rs1554606	G>T	411 (32.2)	616 (48.3)	248 (19.5)
*CXCL8* (chemokine (C‐X‐C motif) ligand 8)	Delirium (van Munster et al. 2011)	rs4073	T>A	357 (28.9)	600 (48.5)	280 (22.6)
*IL10* (interleukin 10)	Neurodegeneration (Arosio et al. 2010)	rs1800872	C>A	728 (57.7)	461 (36.5)	73 (5.8)
		rs1800896	A>G	398 (31.3)	577 (45.4)	297 (23.3)
*IL12B* (interleukin 12B)	Inflammation impact on cognitive function (Goldstein et al. 2014)	rs1368439	T>G	836 (65.7)	397 (31.2)	39 (3.1)
*IL13* (interleukin 13)	Inflammation impact on cognitive function (Goldstein et al. 2014)	rs1800925	C>T	836 (67.2)	364 (29.3)	44 (3.5)
*IL18* (interleukin 18)	Neurodegeneration (Alboni et al. 2010)	rs360729	T>A	609 (48.6)	529 (42.2)	115 (9.2)
		rs5744256	T>C	732 (57.8)	464 (36.7)	70 (5.5)
		rs2043055	A>G	526 (40.9)	585 (45.5)	176 (13.7)
		rs187238	G>C	674 (54.0)	484 (38.8)	91 (7.3)
		rs1946519	C>A	458 (36.3)	592 (46.9)	213 (16.9)
*IGF1* (insulin‐like growth factor 1)	Cognitive dysfunction (Licht et al. 2014)	rs11111272	C>G	648 (51.5)	514 (40.8)	97 (7.7)
		rs10735380	A>G	651 (52.2)	503 (40.3)	94 (7.5)
*IFNGR1* (interferon gamma receptor 1)	Depression, cognitive dysfunction related to aging (Oxenkrug 2011)	rs7749390	A>G	441 (35.2)	621 (49.5)	192 (15.3)
*IFNG* (interferon gamma)	Depression, cognitive dysfunction related to aging (Oxenkrug 2011)	rs2430561	T>A	380 (29.8)	646 (50.6)	250 (19.6)
*NFKB1A* (nuclear factor of kappa light‐chain gene enhancer in B cells inhibitor, alpha)	Neuroplasticity‐related genes, age and cognitive deficit (Li et al. 2015)	rs696	G>A	501 (39.7)	598 (47.3)	164 (13.0)
*CRP* (C‐reactive protein, pentraxin‐related)	Cognitive decline (Mooijaart et al. 2011)	rs1130864	C>T	574 (45.7)	549 (43.7)	133 (10.6)
		rs1800947	G>C	1090 (87.6)	149 (12.0)	6 (0.5)
*TNF* (tumor necrosis factor)	Attention, mental rotation (Beste et al. 2010)	rs1799964	T>C	793 (63.0)	408 (32.4)	58 (4.6)
		rs1800629	G>A	883 (70.1)	339 (26.9)	38 (3.0)
*TNFRSF1A* (tumor necrosis factor receptor superfamily member 1A)	Attention, mental rotation (Beste et al. 2010)	rs767455	T>C	428 (34.3)	597 (47.8)	224 (17.9)
		rs4149570	G>T	465 (36.5)	589 (46.3)	219 (17.2)
*TNFRSF1B* (tumor necrosis factor receptor superfamily member 1B)	Attention, mental rotation (Beste et al. 2010)	rs496888	A>G	645 (51.1)	532 (42.2)	84 (6.7)
		rs976881	G>A	545 (44.1)	551 (44.5)	141 (11.4)
		rs3397	T>C	517 (41.2)	569 (45.3)	169 (13.5)
		rs1061631	G>A	809 (63.7)	412 (32.4)	50 (3.9)
		rs1061622	T>G	730 (58.4)	450 (36.0)	71 (5.7)
*TGFB1* (transforming growth factor beta 1)	Neurocognitive alterations (Loeys et al. 2005)	rs1800469	C>T	570 (46.4)	527 (42.9)	131 (10.7)
*TGFB2* (transforming growth factor beta 2)	Neurocognitive alterations (Loeys et al. 2005)	rs947712	G>A	495 (39.3)	587 (46.6)	179 (14.2)
		rs1418553	C>T	638 (49.9)	524 (41.0)	117 (9.1)
*TLR2* (toll‐like receptor 2)	Neurodegeneration (Crack and Bray 2007)	rs4696480	T>A	325 (25.5)	633 (49.7)	315 (24.7)
		rs3804100	T>C	1091 (86.7)	164 (13.0)	3 (0.2)
		rs3804099^a^, rs5743708^b^				
*TLR4* (toll‐like receptor 4)	Neurodegeneration (Crack and Bray 2007)	rs4986790^b^				
*TLR5* (toll‐like receptor 5)	Neurodegeneration (Crack and Bray 2007)	rs5744168	C>T	1126 (89.5)	130 (10.3)	2 (0.2)
*GCDH* (glutaryl‐CoA dehydrogenase)	Neurodevelopment in mice (Busanello et al. 2013)	rs11085824	A>G	481 (38.5)	603 (48.3)	165 (13.2)

Link refers to studies that analyzed or suggested a relation between the gene and cognitive function.

The absolute numbers and the frequencies of genotypes are written in the following order: homozygous for the most common allele – heterozygotes – homozygous for the minor allele.

a Excluded due to >25% missing values.

b Single‐nucleotide polymorphisms with allele frequency <5% or with Hardy–Weinberg equilibrium test *P*‐values < 0.0005 were excluded.

*Co‐dominant model*: Significant associations were observed between MMSE scores and the SNPs *HTR3E* rs6443950 (*P* = 0.003), *TACR1* rs881 (*P* = 0.006), and *IL6* rs2069835 (*P* = 0.019) in the discovery sample, but the replication in the validation sample did not confirm the associations (Table 3). When only patients receiving ≥400 mg morphine equivalent dose/day (*n* = 300) were analyzed, significant associations between MMSE scores and SNPs *TNFRSF1B* rs3397, *TLR5* rs5744168, *HTR2A* rs6311, and *ADRA2A* rs11195419 were observed in the discovery sample, but did not reach significance in the validation sample (Table 4). After correction for multiple testing, no SNPs were significant in the discovery sample.

**Table 3:** SNPs associated with MMSE scores (*n* = 1369)

Gene	SNP	Minor allele	Discovery sample (*n* = 911)						*P*	Validation sample (*n* = 458)						*P*
			Genotype frequency			MMSE score (median)				Genotype frequency			MMSE score (median)			
Co‐dominant																
* HTR3E*	rs6443950	A	AA	AT	TT	AA	AT	TT	0.003	AA	AT	TT	AA	AT	TT	0.715
			111	402	361	27	28	28		49	227	167	28	28	28	
			CC	CG	GG	CC	CG	GG		CC	CG	GG	CC	CG	GG	
* TACR1*	rs881	C	28	244	607	28.5	28	28	0.006	19	122	304	27	28	28	0.911
			CC	CT	TT	CC	CT	TT		CC	CT	TT	CC	CT	TT	
* IL6*	rs2069835	C	6	103	708	30	28	28	0.019	3	52	354	29	28	27.5	0.472
Dominant																
* HTR3E*	rs6443950	A	AA+AT		TT	AA+AT		TT	0.003	AA+AT		TT	AA+AT		TT	0.658
			763		111	28		27		276		167	28		28	
			TT+CT		CC	TT+CT		CC		TT+CT		CC	TT+CT		CC	
* IL6*	rs2069835	C	811		6	28		30	0.006	55		354	28		28	0.450
			TT+CT		CC	TT+CT		CC		TT+CT		CC	TT+CT		CC	
* HTR2A*	rs6311	T	601		273	28		28	0.019	291		151	28		28	0.594
Recessive																
* TGFB2*	rs1418553	T	CC+CT		TT	CC+CT		TT	0.020	CC+CT		TT	CC+CT		TT	0.666
			765		83	28		27		397		34	28		28.5	
			GG+AG		AA	GG+AG		AA		GG+AG		AA	GG+AG		AA	
* GABBR2*	rs2304389	A	243		613	28		28	0.035	410		20	28		29	0.630
			CG+GG		CC	CG+GG		CC		CG+GG		CC	CG+GG		CC	
* TACR1*	rs881	C	272		607	28		28	0.041	426		19	28		27	0.486

SNP, Single‐nucleotide polymorphisms; MMSE, Mini‐Mental State Examination.

**Table 4:** SNPs associated with MMSE score among patients receiving daily oral morphine equivalent doses of 400 mg or more (*n* = 300)

Gene	SNP	Minor allele	Discovery sample (*n* = 202)						*P*	Validation sample (*n* = 98)						*P*
			Genotype frequency			MMSE score (median)				Genotype frequency			MMSE score (median)			
Co‐dominant																
* TNFRSF1B*	rs3397	C	CC	CT	TT	CC	CT	TT	0.014	CC	CT	TT	CC	CT	TT	0.118
			31	85	75	27	26	28		10	34	50	23.5	28	28.5	
			TC	CT	TT	TC	CT	TT		TC	CT	TT	TC	CT	TT	
* TLR5*	rs5744168	T	179	16	0	27	25	–	0.020	82	10	2	28	26.5	29.5	0.332
			CC	CT	TT	CC	CT	TT		CC	CT	TT	CC	CT	TT	
* HTR2A*	rs6311	T	61	103	31	28	26	27	0.032	29	49	18	27	28	28	0.598
			AA	AC	CC	AA	AC	CC		AA	AC	CC	AA	AC	CC	
* ADRA2A*	rs11195419	A	2	44	133	27.5	28	27	0.039	2	26	65	28	28	28	0.930
Dominant																
* TACR1*	rs2160652	T	TT+GT		GG	TT+GT		GG	0.040	TT+GT		GG	TT+GT		GG	0.523
			106		92	28		26		50		46	28		28	
			TT+CT		CC	TT+CT		CC		TT+CT		CC	TT+CT		CC	
* HTR2A*	rs6311	T	134		61	27		28	0.024	67		29	28		27	0.455
			TT+CT		CC	TT+CT		CC		TT+CT		CC	TT+CT		CC	
* TLR5*	rs5744168	T	16		179	25		27	0.020	12		82	28.5		28	0.982
			AA+AC		CC	AA+AC		CC		AA+AC		CC	AA+AC		CC	
* ADRA2A*	rs11195419	A	46		133	28		27	0.011	28		65	28		28	0.816
			CC+CT		TT	CC+CT		TT		CC+CT		TT	CC+CT		TT	
* TNFRSF1B*	rs3397	C	116		75	26.5		28	0.005	44		50	28		28.5	0.159
*Recessive*																
* GABBR2*	rs2779562	T	CC+CT		TT	CC+CT		TT	0.038	CC+CT		TT	CC+CT		TT	0.656
			141		52	27		28		67		28	28		28	
			AT+TT		AA	AT+TT		AA		AT+TT		AA	AT+TT		AA	
* HTR3E*	rs6443950	A	176		23	27		25	0.034	89		278	28		28.5	0.652
			CC+CT		TT	CC+CT		TT		CC+CT		TT	CC+CT		TT	
* IL6*	rs2069835	T	189		2	27		30	0.029	91		1	28		30	0.188
			CC+CT		TT	CC+CT		TT		CC+CT		TT	CC+CT		TT	
* ADRA2A*	rs553668	T	140		5	28		23	0.022	67		2	28		27	0.731

SNP, Single‐nucleotide polymorphisms; MMSE, Mini‐Mental State Examination.
*Dominant model*: Three significant associations were observed between MMSE scores and SNPs in the discovery sample (*HTR3E* rs*6443950*,* IL6* rs*2069835*, and *HTR2A* rs6311), but the replication in the validation sample did not confirm the associations (Table 3). In patients receiving ≥400 mg morphine equivalent dose/day, there were five significant SNPs in the discovery sample (*TACR1* rs*2160652, HTR2A* rs*6311, TLR5* rs*5744168, ADRA2A* rs*11195419, TNFRSF1B* rs*3397*), but none of them was significant in the validation sample (Table 4). After correction for multiple testing, no SNPs were significant in the discovery sample.
*Recessive model*: Three significant associations were observed between MMSE scores and SNPs in the discovery sample (*TGFB2* rs1418553, *GABBR2* rs2304389, *TACR1* rs881), but the replication in the validation sample did not confirm the associations (Table 3). In patients receiving ≥400 mg morphine equivalent dose/day, there four significant SNPs in the discovery sample (*GABBR2* rs2779562, *HTR3E* rs6443950, *IL6* rs2069835, *ADRA2A* rs553668), but none of them was significant in the validation sample (Table 4). After correction for multiple testing, no SNPs were significant in the discovery sample.

## Discussion

In this study, a thorough exploration of 83 SNPs in 35 genes related to cognitive function was performed using three current well‐accepted genetic models (dominant, co‐dominant and recessive) with discovery (discovery sample) and replication (validation sample) analyses (Lettre et al. 2007). Associations between SNPs and cognitive function in the total sample were explored, as well as considering that opioid can interfere on cognitive function, an analysis of SNPs and cognitive function in those patients receiving ≥400 mg morphine equivalent dose/day was also performed. Although some SNPs were associated with cognitive function in the discovery analysis, the replication did not confirm any associations.

The absence of associations in this study may be due to one or more of the following possibilities: (1) the candidate genes of this study do not interfere with cognitive function; (2) cognitive dysfunction is influenced by polygenic genetic variations instead of isolated SNPs; (3) study limitations including influence of other variables (e.g., medication, comorbidities, general comprehensive measure of cognitive assessment as opposed to several instruments that investigate different specific domains), predefined genes, analysis of different opioids converted as morphine equivalents, and small sample size.

### Targeting the correct genes and analysis approach

The genetic variability and associations with cognitive function is better described in the literature when focusing on specific mental diseases, in which there is a more straightforward identification of impairment and a direct relationship between genetic alteration (usually a mutation) and phenotype. The effect size of common SNPs is generally low and the majority is located in noncoding regions. Any effect from SNPs outside coding regions may be due to linkage disequilibrium with other functional SNPs with higher effect size, but very often at much lower frequency (Edwards et al. 2013).

Moreover, the selection of the analysis methods seems to play a fundamental role. The investigation of genetic variability in the phenotype of interest is usually based on two approaches. In the first approach, a selected number of genetic variations are tested for single associations founded in hypotheses regarding biological functions of candidate genes (candidate gene design). In the second, many random SNPs are tested for associations with phenotype under a statistical correction for multiple hypotheses testing based on the proposition that cognitive traits are controlled by multiple genes (genome‐wide association study) (Rietveld et al. 2014). Until now, these approaches on cognitive function have failed to replicate findings or have found small significant associations (Chabris et al. 2012; Payton 2009; Davies et al. 2011; Benyamin et al. 2014).

In the candidate gene design, most effects of genes on cognitive processing are often analyzed by methods of genetic linkage and association, which result in a statistical modeling that examines relations between a part of the chromosome and a phenotype (Flint 1999). However, it has been suggested that cognitive impairment does not result from a mutation in a single gene and that variations regarding intelligence quotients involve combinations of a number of genes (polygenic genetic basis) that influence, for example, impairment (Nokelainen and Flint 2002). Thus, genome‐wide studies have demonstrated the influence of polygenic variations on cognitive function, psychiatric diseases, and dementing processes (Bulayeva et al. 2015).

Meta‐analysis of population cohorts is another approach in the genome‐wide studies, which can include polygenic analyses. However, the studies showed that the SNPs assessed have accounted for a very small portion (2%) of the phenotypic variance (Rietveld et al. 2013; Davies et al. 2015). Other methods to refine genetic analysis include analysis of subgroups with common characteristics pertinent to specific diseases (Debette et al. 2015). Therefore, the genome‐wide studies have indicated that cognitive dysfunction may result from combination of genetic variants rather than individual effect of a SNP. However, combination of genetic variants often requires large samples in order to successfully replicate findings, estimate predictors by polygenic analyses (Dudbridge 2013), and identify 1–2% of genetic variability. It is a notion for power calculation and estimates of the possible effect sizes of future studies.

### Candidate genes for opioid effects and consequences for cognitive function

Previous knowledge on the association between high opioid doses and cognitive dysfunction (Kurita et al. 2011), and a possible connection with genes that may have influence on opioid effects (Somogyi et al. 2007; Klepstad 2010; Barratt et al. 2014) prompted us to analyze a subgroup of patients receiving high opioid doses. We expected that associations between cognitive dysfunction and SNPs in genes of patients treated with morphine equivalent doses ≥400 mg/day could be found; however, that proved not to be the case. A too small sample size based on a reduced number of patients on high opioid doses may have played a role for the negative outcomes. It is interesting to note that a former study regarding opioid efficacy in the total sample of opioid‐treated patients in the EPOS study did not show significant associations between genetic variability and opioid dosage (Klepstad et al. 2011).

### Strengths and limitations

Strengths of this study include large sample size, diversity of included patients with cancer on opioid treatment, investigation of genes that were reported by the literature to have some relationship with cognitive function, and robust methods of analysis involving three genetic models and removal of false positives. On the other hand, several factors may have hampered the identification of genetic variability related to cognitive function. First, the mechanisms influencing cognitive function are complex and many variables such as medication, psychiatric/psychological disorders, and disease may influence the performance on different neuropsychological tests not related to genetic variation (Kendler and Neale 2010). Second, there exist several neuropsychological tests to assess different cognitive domains and consensus regarding the best instrument for each domain in this particular population is still under development. In this study MMSE was selected due to brevity and easy application, extensive use in research and clinical practice (Folstein et al. 1984; Crum et al. 1993), and its status as the “golden standard” instrument to measure cognitive function in patients with cancer (Meyers and Wefel 2003). However, criticism of MMSE includes rough measurement properties of cognition and psychometric limitations in nondemented populations. The main instrument weaknesses are lack of sensitiveness to detect milder alterations, no contemplation of other important cognitive domains (e.g., executive function), potential learning effect, and influence of other variables as age, schooling, and social background on the score (Spencer et al. 2013). Third, the genes were selected from a pre‐established pool, which did not necessarily encompass all genes potentially associated with cognitive function as APOE, which is associated to Alzheimer's disease and cognitive decline in older age (Ertekin‐Taner 2007; Christensen et al. 2008). Fourth, the different opioids were converted to doses of morphine equivalents in order to allow us to work with a larger sample; however, there is a possibility that each distinct type of opioid (e.g., morphine, oxycodone, fentanyl, and methadone) has a specific interference with cognitive functioning. Fifth, in spite of the large number of patients in the sample, it may not have been large enough to identify significant associations, especially if compared to the modest findings in genome‐wide studies with larger samples. Although our candidate gene approach does not capture all genes and genetic variants that are relevant for cognitive function, applying a genome‐wide association approach was not a realistic option for our study because of the limited sample size and the high threshold for reaching the genome‐wide level of statistical significance. Moreover, the validation sample was smaller than the discovery sample, disregarding any overestimation of effect size (Bush and Moore 2012). The same rationale applies even more pronounced to the analysis of SNPs in patients on high opioid doses (≥400 mg morphine equivalent dose/day) that may also be hampered by the small number of individuals in this subgroup. The effect size of phenotypic characteristics is usually small, which requires analysis of even larger sample sizes (Debette et al. 2015). Nevertheless, small effect size characterizes the veracity of common genetic variability (Edwards et al. 2013).

This study focused on the effects of SNPs on cognitive function of opioid‐treated patients with cancer, and since factors as socio‐demographics, comorbidities, and treatments, among others have been previously explored (Kurita et al. 2011, 2015), they were not reanalyzed. We did not discard the possibility of other variables to interfere with cognitive functioning and overlap genetic interference potentializing the effects or overshadow genetic interference. Prevalent determinants in cancer as aging and inflammation may play an import role. Inflammatory biomarkers have been identified in several neurological diseases (e.g., Parkinson and dementias) and in acute infections, which have been associated with declined cognitive performance (Simen et al. 2011). Also, investigation of inflammatory biomarker levels in African Americans and Caucasians have suggested associations between IL‐8, cognitive function and ethnic background (Goldstein et al. 2014). Moreover, protective measures as intake of nonsteroidal anti‐inflammatory drugs seem to slow down development of neurological diseases as Alzheimer's disease and prevent cognitive decline in subjects with apolipoprotein E (APOE) e4 alleles (Hayden et al. 2007; Gorelick 2010).

Therefore, suggestions for future research in this area should consider the multifactorial nature of cognitive dysfunction and a proper study design. A better understanding of the issue, besides involving genetic aspects (exploration of other sets of genes, combined genes effects, mRNA levels, and polygenic analyses), should also consider several other variables related to cancer. The variety of potential causes for cognitive dysfunction includes known variables in the cancer population (e.g., socio‐demographics, comorbidities, treatment, etc.), information from other conditions (e.g., inflammation biomarkers, dementia structural brain changes, neurodegeneration in older age, etc.), and variables not explored, but involving a plausible hypothesis (e.g., genes analyzed in animal studies). In addition, larger cohorts with adequate sample size and better methods of cognitive assessment are essential to provide high‐quality data and possible definite answers.

In conclusion, the findings of this study did not support influence of those SNPs analyzed to explain cognitive dysfunction in this sample of patients. Several factors may have played a role blurring the potential identification of significant associations. Nonetheless, to the best of our knowledge, this is the first study to explore genetic variability and cognitive dysfunction in opioid‐treated patients with cancer. Larger multicenter collaboration and interest of funding institutions are highly required for further investigation.

## Conflict of Interest

None declared.

## References

[brb3471-bib-0001] Alboni, S. , D. Cervia , S. Sugama , and B. Conti . 2010 Interleukin 18 in the CNS. J. Neuroinflammation 7:9. 20113500 10.1186/1742-2094-7-9 PMC2830964

[brb3471-bib-0002] Arosio, B. , L. Mastronardi , C. Vergani , and G. Annoni . 2010 Intereleukin‐10 promoter polymorphism in mild cognitive impairment and in its clinical evolution. Int. J. Alzheimers Dis. pii:854527. 20798845 10.4061/2010/854527 PMC2925379

[brb3471-bib-0003] Barratt, D. T. , B. Bandak , P. Klepstad , O. Dale , S. Kaasa , L. L. Christrup , et al. 2014 Genetic, pathological and physiological determinants of transdermal fentanyl pharmacokinetics in 620 cancer patients of the EPOS study. Pharmacogenet. Genomics 24:185–194. 24469018 10.1097/FPC.0000000000000032

[brb3471-bib-0004] Baumgartner, K. 2004 Neurocognitive changes in cancer patients. Semin. Oncol. Nurs. 20:284–290. 15612604

[brb3471-bib-0005] Benjamini, Y. , and Y. Hochberg . 1995 Controlling the false discovery rate: a practical and powerful approach to multiple testing. J. R. Stat. Soc. B 57:289–300.

[brb3471-bib-0006] Benke, K. S. , M. C. Carlson , B. Q. Doan , J. D. Walston , Q. L. Xue , A. P. Reiner , et al. 2011 The association of genetic variants in interleukin‐1 genes with cognition: findings from the cardiovascular health study. Exp. Gerontol. 46:1010–1019. 21968104 10.1016/j.exger.2011.09.005 PMC3689225

[brb3471-bib-0007] Benyamin, B. , B. Pourcain , O. S. Davis , G. Davies , N. K. Hansell , M. J. Brion , et al. 2014 Childhood intelligence is heritable, highly polygenic and associated with FNBP1L. Mol. Psychiatry 19:253–258. 23358156 10.1038/mp.2012.184 PMC3935975

[brb3471-bib-0008] Beste, C. , M. Heil , K. Domschke , B. T. Baune , and C. Konrad . 2010 Associations between the tumor necrosis factor alpha gene (‐308G→A) and event‐related potential indices of attention and mental rotation. Neuroscience 170:742–748. 20682333 10.1016/j.neuroscience.2010.07.058

[brb3471-bib-0009] Bochdanovits, Z. , F. M. Gosso , L. van den Berg , P. Rizzu , T. J. Polderman , L. M. Pardo , et al. 2009 A functional polymorphism under positive evolutionary selection in ADRB2 is associated with human intelligence with opposite effects in the young and the elderly. Behav. Genet. 39:15–23. 18855131 10.1007/s10519-008-9233-0

[brb3471-bib-0010] Bruera, E. , L. Miller , J. McCallion , K. Macmillan , L. Krefting , and J. Hanson . 1992 Cognitive failure in patients with terminal cancer: a prospective study. J. Pain Symptom Manage. 7:192–195. 1517640 10.1016/0885-3924(92)90074-r

[brb3471-bib-0011] Bulayeva, K. , K. P. Lesch , O. Bulayev , C. Walsh , S. Glatt , F. Gurgenova , et al. 2015 Genomic structural variants are linked with intellectual disability. J. Neural. Transm. (Vienna). 122:1289–1301. 25626716 10.1007/s00702-015-1366-8 PMC4517986

[brb3471-bib-0012] Bullido, M. J. , M. C. Ramos , A. Ruiz‐Gómez , A. S. Tutor , I. Sastre , A. Frank , et al. 2004 Polymorphism in genes involved in adrenergic signaling associated with Alzheimer's. Neurobiol. Aging 25:853–859. 15212839 10.1016/j.neurobiolaging.2003.10.006

[brb3471-bib-0013] Busanello, E. N. , L. Pettenuzzo , P. H. Botton , P. Pandolfo , D. O. de Souza , M. Woontner , et al. 2013 Neurodevelopmental and cognitive behavior of glutaryl‐CoA dehydrogenase deficient knockout mice. Life Sci. 92:137–142. 23201428 10.1016/j.lfs.2012.11.013

[brb3471-bib-0014] Bush, W. S. , and J. H. Moore . 2012 Chapter 11: genome–wide association studies. PLoS Comput. Biol. 8:e1002822. 23300413 10.1371/journal.pcbi.1002822 PMC3531285

[brb3471-bib-0016] Chabris, C. F. , B. M. Hebert , D. J. Benjamin , J. Beauchamp , D. Cesarini , M. van der Loos , et al. 2012 Most reported genetic associations with general intelligence are probably false positives. Psychol. Sci. 23:1314–1323. 23012269 10.1177/0956797611435528 PMC3498585

[brb3471-bib-0017] Christensen, H. , P. J. Batterham , A. J. Mackinnon , A. F. Jorm , H. A. Mack , K. A. Mather , et al. 2008 The association of APOE genotype and cognitive decline in interaction with risk factors in a 65–69 year old community sample. BMC Geriatr. 8:14. 18620605 10.1186/1471-2318-8-14 PMC2488328

[brb3471-bib-0018] Crack, P. J. , and P. J. Bray . 2007 Toll‐like receptors in the brain and their potential roles in neuropathology. Immunol. Cell Biol. 85:476–480. 17667932 10.1038/sj.icb.7100103

[brb3471-bib-0019] Crum, R. M. , J. C. Anthony , S. S. Bassett , and M. F. Folstein . 1993 Population‐based norms for the Mini‐Mental State Examination by age and educational level. JAMA 269:2386–2391. 8479064

[brb3471-bib-0020] Dai, H. , K. Kaneko , H. Kato , S. Fujii , Y. Jing , A. Xu , et al. 2007 Selective cognitive dysfunction in mice lacking histamine H1 and H2 receptors. Neurosci. Res. 57:306–313. 17145090 10.1016/j.neures.2006.10.020

[brb3471-bib-0021] Davies, G. , A. Tenesa , A. Payton , J. Yang , S. E. Harris , D. Liewald , et al. 2011 Genome‐wide association studies establish that human intelligence is highly heritable and polygenic. Mol. Psychiatry 16:996–1005. 21826061 10.1038/mp.2011.85 PMC3182557

[brb3471-bib-0022] Davies, G. , N. Armstrong , J. C. Bis , J. Bressler , V. Chouraki , S. Giddaluru , et al. 2015 Genetic contributions to variation in general cognitive function: a meta‐analysis of genome‐wide association studies in the CHARGE consortium (N = 53 949). Mol. Psychiatry 20:183–192. 25644384 10.1038/mp.2014.188 PMC4356746

[brb3471-bib-0023] Debette, S. , C. A. Ibrahim Verbaas , J. Bressler , M. Schuur , A. Smith , J. C. Bis , et al. 2015 Genome‐wide studies of verbal declarative memory in nondemented older people: the cohorts for heart and aging research in Genomic Epidemiology Consortium. Biol. Psychiatry 77:749–763. 25648963 10.1016/j.biopsych.2014.08.027 PMC4513651

[brb3471-bib-0024] Dudbridge, F. 2013 Power and predictive accuracy of polygenic risk scores. PLoS Genet. 9:e1003348. 23555274 10.1371/journal.pgen.1003348 PMC3605113

[brb3471-bib-0025] Edwards, S. L. , J. Beesley , J. D. French , and A. M. Dunning . 2013 Beyond GWASs: illuminating the dark road from association to function. Am. J. Hum. Genet. 93:779–797. 24210251 10.1016/j.ajhg.2013.10.012 PMC3824120

[brb3471-bib-0026] Ertekin‐Taner, N. 2007 Genetics of Alzheimer's disease: a centennial review. Neurol. Clin. 25:611–667. 17659183 10.1016/j.ncl.2007.03.009 PMC2735049

[brb3471-bib-0027] Fiocco, A. J. , K. Lindquist , R. Ferrell , R. Li , E. M. Simonsick , M. Nalls , et al. 2010 COMT genotype and cognitive function: an 8‐year longitudinal study in white and black elders. Neurology 74:1296–1302. 20404311 10.1212/WNL.0b013e3181d9edba PMC2860484

[brb3471-bib-0029] Flint, J. 1999 The genetic basis of cognition. Brain 122:2015–2031. 10545388 10.1093/brain/122.11.2015

[brb3471-bib-0030] Flint, J. 2001 Genetic basis of cognitive disability. Dialogues Clin. Neurosci. 3:37–46. 22034445 10.31887/DCNS.2001.3.1/jflint PMC3181642

[brb3471-bib-0031] Folstein, M. F. , J. H. Fetting , A. Lobo , U. Niaz , and K. D. Capozzoli . 1984 Cognitive assessment of cancer patients. Cancer 53:2250–2257. 6704913 10.1002/cncr.1984.53.s10.2250

[brb3471-bib-0033] Gizer, I. R. , C. Ficks , and I. D. Waldman . 2009 Candidate gene studies of ADHD: a meta‐analytic review. Hum. Genet. 126:51–90. 19506906 10.1007/s00439-009-0694-x

[brb3471-bib-0034] Goldstein, F. C. , L. Zhao , K. Steenland , and A. I. Levey . 2014 Inflammation and cognitive functioning in African Americans and Caucasians. Int. J. Geriatr. Psychiatry 30:934–941. 25503371 10.1002/gps.4238 PMC4682666

[brb3471-bib-0035] Gorelick, P. B. 2010 Role of inflammation in cognitive impairment: results of observational epidemiological studies and clinical trials. Ann. N. Y. Acad. Sci. 1207:155–162. 20955439 10.1111/j.1749-6632.2010.05726.x

[brb3471-bib-0037] Hayden, K. M. , P. P. Zandi , A. S. Khachaturian , C. A. Szekely , M. Fotuhi , M. C. Norton , et al. 2007 Does NSAID use modify cognitive trajectories in the elderly? The Cache County study. Neurology 69:275–282. 17636065 10.1212/01.wnl.0000265223.25679.2a

[brb3471-bib-0038] Inouye, S. K. , M. D. Foreman , L. C. Mion , K. H. Katz , and L. M. Jr Cooney . 2001 Nurses' recognition of delirium and its symptoms: comparison of nurse and researcher ratings. Arch. Intern. Med. 161:2467–2473. 11700159 10.1001/archinte.161.20.2467

[brb3471-bib-0039] Kendler, K. S. , and M. C. Neale . 2010 Endophenotype: a conceptual analysis. Mol. Psychiatry 15:789–797. 20142819 10.1038/mp.2010.8 PMC2909487

[brb3471-bib-0040] Klepstad, P. 2010 Pharmacogenetic considerations in the treatment of cancer pain Pp. 180–194 in Bruera E. and Portenoy R. K., eds. Cancer pain. Cambridge Univ. Press, New York, NY.

[brb3471-bib-0041] Klepstad, P. , T. Fladvad , F. Skorpen , K. Bjordal , A. Caraceni , O. Dale , et al. 2011 Influence from genetic variability on opioid use for cancer pain: a European genetic association study of 2294 cancer pain patients. Pain 152:1139–1145. 21398039 10.1016/j.pain.2011.01.040

[brb3471-bib-0042] Kurita, G. P. , and C. A. M. Pimenta . 2008 Cognitive impairment in cancer pain patients receiving opioids: a pilot study. Cancer Nurs. 31:49–57. 18176132 10.1097/01.NCC.0000305673.06211.cd

[brb3471-bib-0043] Kurita, G. P. , L. Lundorff , C. A. M. Pimenta , and P. Sjøgren . 2009 The cognitive effects of opioids in cancer: a systematic review. Support. Care Cancer 17:11–21. 18762994 10.1007/s00520-008-0497-y

[brb3471-bib-0044] Kurita, G. P. , P. Sjøgren , O. Ekholm , S. Kaasa , J. H. Loge , I. Poviloniene , et al. 2011 Prevalence and predictors of cognitive dysfunction in opioid treated cancer patients: a multinational study. J. Clin. Oncol. 29:1297–1303. 21357785 10.1200/JCO.2010.32.6884

[brb3471-bib-0045] Kurita, G. P. , S. Lundström , P. Sjøgren , O. Ekholm , L. Christrup , A. Davies , et al. 2015 Renal function and symptoms/adverse effects in opioid‐treated patients with cancer. Acta Anaesthesiol. Scand. 59:1049–1059. 25943005 10.1111/aas.12521

[brb3471-bib-0046] Lawlor, P. G. 2002 The panorama of opioid‐related cognitive dysfunction in patients with cancer. Cancer 94:1836–1853. 11920548 10.1002/cncr.10389

[brb3471-bib-0047] Lettre, G. , C. Lange , and J. N. Hirschhorn . 2007 Genetic model testing and statistical power in population‐based association studies of quantitative traits. Genet. Epidemiol. 31:358–362. 17352422 10.1002/gepi.20217

[brb3471-bib-0048] Levine, P. M. , P. M. Silberfarb , and Z. J. Lipowski . 1978 Mental disorders in cancer patients: a study of 100 psychiatric referrals. Cancer 42:1385–1391. 698920 10.1002/1097-0142(197809)42:3<1385::aid-cncr2820420349>3.0.co;2-0

[brb3471-bib-0049] Li, Y. , A. Abdourahman , J. A. Tamm , A. L. Pehrson , C. Sánchez , and M. Gulinello . 2015 Reversal of age‐associated cognitive deficits is accompanied by increased plasticity‐related gene expression after chronic antidepressant administration in middle‐aged mice. Pharmacol. Biochem. Behav. 135:70–82. 26046533 10.1016/j.pbb.2015.05.013

[brb3471-bib-0050] Licht, C. M. , L. C. van Turenhout , J. B. Deijen , L. L. Koppes , W. van Mechelen , J. W. Twisk , et al. 2014 The association between IGF‐1 polymorphisms, IGF‐1 serum levels, and cognitive functions in healthy adults: the Amsterdam Growth and Health Longitudinal Study. Int. J. Endocrinol. 2014:181327. 25114679 10.1155/2014/181327 PMC4120488

[brb3471-bib-0051] Loeys, B. L. , J. Chen , E. R. Neptune , D. P. Judge , M. Podowski , T. Holm , et al. 2005 A syndrome of altered cardiovascular, craniofacial, neurocognitive and skeletal development caused by mutations in TGFBR1 or TGFBR2. Nat. Genet. 37:275–281. 15731757 10.1038/ng1511

[brb3471-bib-0052] Massie, M. J. , J. Holland , and E. Glass . 1983 Delirium in terminally ill cancer patients. Am. J. Psychiatry 140:1048–1050. 6869591 10.1176/ajp.140.8.1048

[brb3471-bib-0053] Meneses, A. 1999 5‐HT system and cognition. Neurosci. Biobehav. Rev. 23:1111–1125. 10643820 10.1016/s0149-7634(99)00067-6

[brb3471-bib-0054] Meyers, C. A. , and J. S. Wefel . 2003 The use of the Mini‐Mental State Examination to assess cognitive functioning in cancer trails: no, ifs, ands, buts, or sensitivity. J. Clin. Oncol. 21:3557–3558. 12913103 10.1200/JCO.2003.07.080

[brb3471-bib-0055] Mooijaart, S. P. , N. Sattar , S. Trompet , E. Polisecki , A. J. de Craen , E. J. Schaefer , et al. 2011 C‐reactive protein and genetic variants and cognitive decline in old age: the PROSPER study. PLoS One 6:e23890. 21915265 10.1371/journal.pone.0023890 PMC3168438

[brb3471-bib-0056] van Munster, B. C. , A. H. Zwinderman , and S. E. de Rooij . 2011 Genetic variations in the interleukin‐6 and interleukin‐8 genes and the interleukin‐6 receptor gene in delirium. Rejuvenation Res. 14:425–428. 21851175 10.1089/rej.2011.1155

[brb3471-bib-0057] Nokelainen, P. , and J. Flint . 2002 Genetic effects on human cognition: lessons from the study of mental retardation syndromes. J. Neurol. Neurosurg. Psychiatry 72:287–296. 11861682 10.1136/jnnp.72.3.287 PMC1737778

[brb3471-bib-0058] Oxenkrug, G. 2011 Interferon‐gamma – inducible inflammation: contribution to aging and aging‐associated psychiatric disorders. Aging Dis. 2:474–486. 22396896 PMC3295064

[brb3471-bib-0059] Payton, A. 2009 The impact of genetic research on our understanding of normal cognitive ageing: 1995 to 2009. Neuropsychol. Rev. 19:451–477. 19768548 10.1007/s11065-009-9116-z

[brb3471-bib-0061] Pisani, M. A. , C. Redlich , L. McNicoll , E. W. Ely , and S. K. Inouye . 2003 Under‐ recognition of preexisting cognitive impairment by physicians in older ICU patients. Chest 124:2267–2274. 14665510 10.1378/chest.124.6.2267

[brb3471-bib-0062] Rietveld, C. A. , S. E. Medland , J. Derringer , J. Yang , T. Esko , N. W. Martin , et al. 2013 GWAS of 126,559 individuals identifies genetic variants associated with educational attainment. Science 340:1467–1471. 23722424 10.1126/science.1235488 PMC3751588

[brb3471-bib-0063] Rietveld, C. A. , T. Esko , G. Davies , T. H. Pers , P. Turley , B. Benyamin , et al. 2014 Common genetic variants associated with cognitive performance identified using the proxy‐phenotype method. Proc. Natl Acad. Sci. USA 111:13790–13794. 25201988 10.1073/pnas.1404623111 PMC4183313

[brb3471-bib-0064] Sasayama, D. , H. Hori , T. Teraishi , K. Hattori , M. Ota , J. Matsuo , et al. 2011 Association of interleukin‐1*β* genetic polymorphisms with cognitive performance in elderly females without dementia. J. Hum. Genet. 56:613–616. 21614008 10.1038/jhg.2011.56

[brb3471-bib-0065] Simen, A. A. , K. A. Bordner , M. P. Martin , L. A. Moy , and L. C. Barry . 2011 Cognitive dysfunction with aging and the role of inflammation. Ther. Adv. Chronic. Dis. 2:175–195. 23251749 10.1177/2040622311399145 PMC3513880

[brb3471-bib-0066] Sjøgren, P. 1997 Psychomotor and cognitive functioning in cancer patients. Acta Anaesthesiol. Scand. 41:159–161. 9061100 10.1111/j.1399-6576.1997.tb04631.x

[brb3471-bib-0067] Somogyi, A. , D. T. Barratt , and J. K. Coller . 2007 Pharmacogenetics of opioids. Clin. Pharmacol. Ther. 81:429–444. 17339873 10.1038/sj.clpt.6100095

[brb3471-bib-0068] Spencer, R. J. , C. R. Wendell , P. P. Giggey , L. I. Katzel , D. M. Lefkowitz , E. L. Siegel , et al. 2013 Psychometric limitations of the mini‐mental state examination among nondemented older adults: an evaluation of neurocognitive and magnetic resonance imaging correlates. Exp. Aging Res. 39:382–397. 23875837 10.1080/0361073X.2013.808109

[brb3471-bib-0070] Wang, X. , W. Sun , X. Zhu , L. Li , X. Wu , H. Lin , et al. 2008 Association between the gamma‐aminobutyric acid type B receptor 1 and 2 gene polymorphisms and mesial temporal lobe epilepsy in a Han Chinese population. Epilepsy Res. 81:198–203. 18653317 10.1016/j.eplepsyres.2008.06.001

[brb3471-bib-0071] World Health Organization . 1996 Cancer pain relief: with a guide to opioid availability, 2nd ed World Health Organization, Singapore.

[brb3471-bib-0072] Yan, T. C. , J. A. Dudley , R. K. Weir , E. M. Grabowska , Y. Peña‐Oliver , T. L. Ripley , et al. 2011 Performance deficits of NK1 receptor knockout mice in the 5‐choice serial reaction‐time task: effects of d‐amphetamine, stress and time of day. PLoS ONE 6:e17586. 21408181 10.1371/journal.pone.0017586 PMC3049786

